# Haul-Out Behavior of Harbor Seals (*Phoca vitulina*) in Hood Canal, Washington

**DOI:** 10.1371/journal.pone.0038180

**Published:** 2012-06-18

**Authors:** Josh M. London, Jay M. Ver Hoef, Steven J. Jeffries, Monique M. Lance, Peter L. Boveng

**Affiliations:** 1 National Marine Mammal Laboratory, Alaska Fisheries Science Center, National Marine Fisheries Service, Seattle, Washington, United States of America; 2 Marine Mammal Investigations, Washington Department of Fish and Wildlife, Tacoma, Washington, United States of America; University of Swansea, United Kingdom

## Abstract

The goal of this study was to model haul-out behavior of harbor seals (*Phoca vitulina*) in the Hood Canal region of Washington State with respect to changes in physiological, environmental, and temporal covariates. Previous research has provided a solid understanding of seal haul-out behavior. Here, we expand on that work using a generalized linear mixed model (GLMM) with temporal autocorrelation and a large dataset. Our dataset included behavioral haul-out records from archival and VHF radio tag deployments on 25 individual seals representing 61,430 seal hours. A novel application for increased computational efficiency allowed us to examine this large dataset with a GLMM that appropriately accounts for temporal autocorellation. We found significant relationships with the covariates hour of day, day of year, minutes from high tide and year. Additionally, there was a significant effect of the interaction term hour of day : day of year. This interaction term demonstrated that seals are more likely to haul out during nighttime hours in August and September, but then switch to predominantly daylight haul-out patterns in October and November. We attribute this change in behavior to an effect of human disturbance levels. This study also examined a unique ecological event to determine the role of increased killer whale (*Orcinus orca*) predation on haul-out behavior. In 2003 and 2005 these harbor seals were exposed to unprecedented levels of killer whale predation and results show an overall increase in haul-out probability after exposure to killer whales. The outcome of this study will be integral to understanding any changes in population abundance as a result of increased killer whale predation.

## Introduction

Harbor seals (*Phoca vitulina*) are one of the most abundant and widespread pinnipeds in the eastern North Pacific Ocean. Throughout their range, harbor seals haul out at near-shore sites on a regular basis and have a moderate level of fidelity to those sites. Seals haul out for a variety of reasons including rest, thermoregulation, predator avoidance, social interaction, molting, pupping and nursing [1]–[5]. The relative importance of these behaviors and physiological functions changes over time and differs between ages and the sexes [6], [7]. Additionally, environmental variables (e.g., tidal state) influence the availability or suitability of preferred haul-out locations. Thus, at any given time, only a portion of the population will be ashore and that proportion changes with respect to these environmental variables, physiological functions and behaviors. The goal of this study was to model the haul-out behavior of harbor seals with respect to changes in demographic, environmental and temporal covariates.

Harbor seal haul-out behavior has been studied by several researchers [7]–[11]. This research has provided a solid understanding of seal behavior and has provided critical information for estimating population status and trends [12]–[15]. Our study expands on previous work in two key areas: 1) the use of a generalized linear mixed model (GLMM) that accounts for temporal auto-correlation with a large dataset to model seal haul-out behavior, 2) the unique opportunity to examine the impact of exposure to increased killer whale (*Orcinus orca*) predation on haul-out probability.

In many of these studies, tidal state is the most consistent factor influencing the daily timing of when seals haul out. With some exceptions (e.g. Hood Canal, Washington), the highest proportion of seals ashore occurs between 2 hours before and 2 hours after low tide. Lower tides often expose rocky reefs, sandy beaches and mudflats that are favorable haul-out sites for seals because of isolation from land predators and quick access to deep water. In areas where seals rest on habitats or man-made structures that are available at all tides, tidal state is less influential.

Temporal cycles also play a role in determining the proportion of seals ashore. Peak counts of seals at haul-out sites typically center on low tides that occur during the middle of the day [4], [13], [16], [17]. When researchers monitored the presence of seals across all hours of the day, most found more seals present during day than night hours [3], [17]. Additionally, the annual breeding and molting cycle of seals leads to temporal changes in haul-out behavior. During pupping and weaning, adult females and pups spend more time ashore [18], whereas males focus on establishing and defending aquatic territories [19], [20]. All age and sex classes, with the exception of pups, go through a complete molt every year. The largest proportion of seals ashore often occurs during the molt period [3], [9], [21]; however, the timing of molt often varies with age and sex [18], [22]. These temporal cycles work in concert with tidal influences to affect the overall pattern of harbor seal haul-out behavior.

Predation risk is an additional factor influencing the time seals spend ashore. Vulnerability to land-based predators (e.g. bears, wolves, coyotes, dogs) likely influences where seals haul out and sites that are isolated from the mainland are often preferred [23]. Hauling out on land also provides seals relative safety from marine predators (i.e., killer whales and sharks). The expectation is that as predation risk from marine predators increases, the amount of time seals spend ashore would also increase until the need for energy outweighs the risk of predation. Unfortunately, quantifying predation risk for inclusion in a model of haul-out behavior has not been possible.

**Figure 1:**
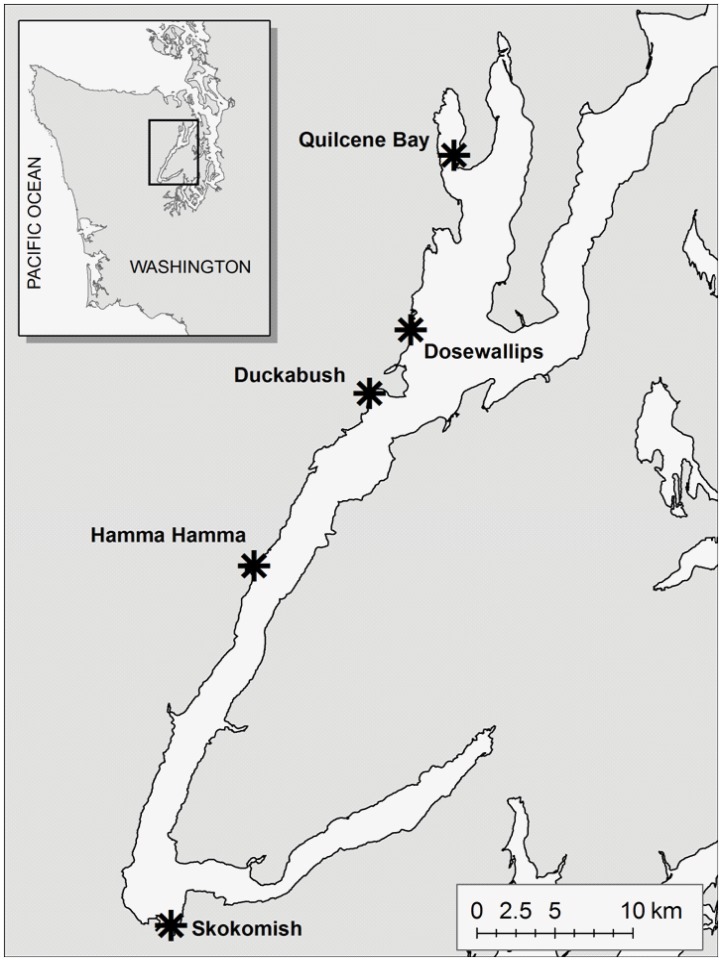
Map of Hood Canal, Washington, showing five major harbor seal haul-out sites.

Hood Canal is a fjord-like body of water located in western Puget Sound of Washington state (Fig. 1). Harbor seals are the most abundant and only resident marine mammal within Hood Canal. In the winter of 2003, 11 mammal-eating [24] killer whales spent 59 days in Hood Canal. Other than a few generic (no ecotype identified) killer whale observations and less than 3 confirmed observations of fish-eating residents, killer whales do not visit Hood Canal with any regularity. A second group of 6 different mammal-eating killer whales was in Hood Canal for a total 172 days in 2005. In both years, bio-energetic models and on-water observations predicted consumption of approximately 1000 seals by these whales within Hood Canal [25]. The dramatic increase in exposure to killer whale predation experienced by harbor seals in Hood Canal provided an opportunity to test the impact of predation on haul-out behavior of seals.

Hood Canal seals are part of the inland Washington stock of harbor seals, but differ significantly in key areas of haul-out timing and pupping phenology. Seals in Hood Canal are more likely to haul out at high tide [26]. Additionally, the pupping and molting periods in Hood Canal appear to extend well into October and early November. For the remainder of the Washington inland stock, peak pupping generally occurs between July and August; molting for the inland stock is in late August and September [14], [18].

Seals haul out at a variety of sites within Hood Canal, but over 85% of the seals counted are found at 5 main haul-out sites: Quilcene Bay, Dosewallips River delta, Duckabush River delta, Hamma Hamma River delta and Skokomish River delta (Fig. 1). With the exception of Quilcene Bay, seals usually haul out on the edge of tidal sloughs. At high tide, these slough-edge habitats provide seals with isolated, level resting areas while also allowing easy access to deep water during periods of high tide. Pilot tracking studies in Hood Canal (concurrent with this study) indicate some level of interchange between haul-out sites and overlap in their use of the marine habitat. The haul-out site in Quilcene Bay is located on floating oyster and salmon net pens, and, as such, tidal state does not affect access by harbor seals.

This study presents data from archival time-depth recorders (TDR) deployed and recovered from harbor seals in Hood Canal during the pupping and early molting periods in 2002 (pre-killer whale exposure) and in 2005 (post-killer whale exposure). Additionally, we present 2006 data from flipper-mounted VHF tags and shore-based VHF data. These data were used to model haul-out behavior of harbor seals in Hood Canal with respect to known demographic (age, sex), environmental (tidal state) and temporal (hour of day, day of year, and year) covariates. General linear models (GLM), general additive models (GAM) and GLMMs can all model this type of binary ‘time-line’ data [12], [17], [27]. The GLMM is advantageous in this scenario because it accounts for temporal auto-correlation and random effects for variability among seals [27]. Previous implementations of GLMMs that account for temporal auto-correlation were limited in computational efficiency and analysis of the long time series that are typical of haul-out data was not possible. In this study, we used a GLMM with an exponential auto-covariance model for repeated measurements to allow efficient analysis of these large data sets [28].

## Materials and Methods

### Ethics Statement

All work was conducted in accordance with and under the authority of the United States Marine Mammal Protection Act (NMFS Permit # 782-1702). The Marine Mammal Protection Act was established in 1972 requiring all research conducted on marine mammals in the United States be done under the authority of federal permits issued by either the National Marine Fisheries Service (NMFS) or U.S. Fish and Wildlife Service (USFWS). All applications for a permit to conduct research on marine mammals have gone through a four-stage review process that includes: 1) agency review (either NMFS or USFWS); 2) a public notice and review period; 3) review and recommendation from the Scientific Advisers to the U.S. Marine Mammal Commission; and 4) a final action by the reviewing agency. All capture and handling activities described in this manuscript have gone through and been approved by this process. At the time this work was conducted there was no additional requirement for review of these procedures by an institutional review board or ethics committee. In 2010, a NMFS Institutional Animal Care and Use Committee (IACUC) was established for the Alaska Fisheries and Northwest Fisheries science centers and the capture and handling protocols described here were reviewed and approved by this committee.

### Study Design

The TDR and VHF deployments were originally conducted as part of a multi-year foraging ecology study of harbor seals in Hood Canal. Given the original study objectives and the unplanned nature of the two killer whale incursions, the design and instrument deployments were not ideal for addressing haul-out behavior and potential influences of killer whale presence and predation. For this study, and the unique ecological opportunity it provides, we have made an effort to mitigate any design concerns by relying on more stringent analysis techniques (e.g. GLMM with temporal auto-correlation) and being transparent regarding our analysis assumptions. Researchers hoping to answer similar questions should attempt more balance between haul-out sites and should consider use of satellite-linked tags that archive and transmit haul-out behavior records via Argos or GSM based systems.

### TDR Deployment

Seals were captured with either a beach seine technique or by using mono-filament tangle nets [29]. In 2002, all seals were captured at the Dosewallips haul-out site. In 2005, most packages were deployed at the Dosewallips site, with a few animals captured at Quilcene Bay, Duckabush and Skokomish haul-out sites.

Archival time-depth recorders (TDR) (model Mk9, Wildlife Computers, Redmond, WA) were attached to all age and sex classes with the exception of pups in Hood Canal during 2002 (n = 16) and 2005 (n = 22). We deployed the majority of TDRs in late July and early August; tags remained attached to the seals as late as 1 November. TDRs were programmed to record an electrical resistance value of the surrounding medium (water or air) every 10 s. The resistance value ranged from 0–255, with values near 0 indicating low resistance (wet) and those near 255 representing high resistance (dry). Each TDR was incorporated into a flotation pack (Pacific Eco-tec, Nanaimo, B.C., Canada) that included an internal VHF transmitter (Advanced Telemetry Systems (ATS), Isanti, MN, USA). The packages were located on the mid-dorsal region, and we used five-minute epoxy to adhere packs to the hair. Once the animals molted (September-October), the TDR packages fell off and were either recovered by researchers using VHF receivers or beachcombers who returned the packages to the Washington Department of Fish and Wildlife. After the tags were recovered, data were downloaded and decoded using software supplied by the manufacturer.

### VHF Deployment

Flipper-mounted VHF transmitters (164–165 mhz) were deployed in 2006 on seals at Skokomish River sites. Seals were captured as in 2002 and 2005, and transmitters were attached to all age and sex classes. An ATS Data Collection Computer (DCC, models D5041A and DCCII) and receiver (model R2100) were installed on a hill above the Skokomish River delta with an unobstructed view of the entire haul-out area. The DCC program scanned each frequency for 10 s. The number of frequencies scanned remained constant over the duration of the deployments; each frequency was scanned 15 times per hour. The DCC used pattern matching to validate reception by matching the pulse rate. All transmitters were set at a pulse rate of 60 pulses per minute and the expected number of pulses recorded by the DCC was between 9 and 11. The number of pulses was significantly greater than expected for a small portion of the data. This was likely due to signal interference from other transmissions in the area. The pattern-matching algorithm detects transmitter reception in the presence of interference and the DCC only logs signals that pass the pattern matching criteria. To increase confidence in the data set, all records for which the recorded number of pulses exceeded 25 or was less than 5 were removed from analysis.

VHF deployments for analysis of haul-out patterns could be confounded because the DCC only detects haul-out bouts within range of the DCC (approx. 8 km line-of-sight). We assume if the DCC did not detect the transmitter frequency, then the seal was not hauled out. In fact, the animal could have moved beyond the range of the DCC and hauled out at a different location. A combination of additional roadside and aerial VHF tracking, as well as additional DCC listening stations, provided some verification that deployed tags remained within range of the Skokomish DCC. A similar approach was employed during the 2005 TDR deployments to verify those seals remained within the Hood Canal region. For the TDR deployments in 2002, captured seals were outfitted with head-mounted VHF tags and were located from a boat at least once a week. For a given seal, any periods of significant absence from the DCC record and additional tracking efforts were removed from analysis.

### Data Preparation

We aggregated records from the TDR and VHF deployments, relevant capture information and various tidal covariates into hourly time-lines. We then truncated TDR records only to include data recorded after release and prior to the last significant dive recorded by the instrument. We grouped the resistance values recorded by TDRs by hour and calculated the hourly average value for each seal. The resistance values range from 0–255 with 0 representing no resistance and, thus, the tag is submerged in salt water. 255 corresponds to maximum resistance and the tag is in the air. To convert the resitance values to a binary statistic, all hourly averages greater than 127 were categorized as hauled out and values 127 or less were categorized as not hauled out. We matched the hourly time-lines with tidal covariates (time from high tide, tidal height) as calculated by the MDR wtides (http://www.wtides.com) software package. The difference in the timing of tidal state between the Dosewallips region and the Skokomish region is approximately 15 minutes. All values were determined based on tidal values at the half-hour for the Triton Head location (47.6033,−122.9817) in central Hood Canal.

For the VHF data, we aggregated the logged detections into hourly time-lines for compatibility with the TDR records. Uninterrupted VHF logging occurred from 14 September through 29 November 2006. Haul-out status was determined by dividing the number of possible detections by the number of times the DCC detected each transmitter for each hour. In those cases where interference required removal of the records from analysis, we adjusted the denominator accordingly. For those hours in which the fraction of scans with confirmed reception exceeded 0.5, we classified those seals as hauled out. Hours with values less than or equal to 0.5 were classified as not hauled out. We matched tidal covariates to the hourly VHF record-set using the same procedure employed for the TDR data.

### GLMM Analysis

We fit a GLMM to the time-line haul-out data for analysis of the relationship between seal haul-out behavior and our covariates. The response variable was binary time-line data with a ‘1′ indicating a seal was mostly hauled out during the hour. We included the following explanatory variables in the model: 1) hour as a categorical variable with 24 levels, 2) minutes from high tide grouped into 30 equally spaced categories for every 32 minutes, with the category midpoints starting at 448 minutes prior to high tide to 482 minutes after high tide, 3) days from 15 August as a continuous variable, 4) year as a categorical variable for 2002, 2005, and 2006, 5) sex as a categorical variable, 6) and age as a categorical variable with levels pup, yearling, sub-adult, and adult. Because the data were binary, we used the logit link and binomial variance function [30], similar to logistic regression. We also included temporal autocorrelation and a random effect for variability among seals. Note that some explanatory variables pertain to the individual seal (sex, year, and age), and some pertain to the repeated measurements of seals (hour, date, and tide). Hence, we used a repeated measurement analysis with temporal autocorrelation on the logit scale because of the binary nature of the data. The opportunistic nature of the study resulted in site and year being confounded (seals from different sites were captured in different years). Ancillary data from tracking studies and behavior observations suggest there is some interchange between haul-out sites in Hood Canal and the haul-out substrates and tidal influences are similar. Additionally, the killer whales exploited habitats throughout Hood Canal so all sites were exposed similarly to any predation threat. For these reasons, we have chosen to include a year effect in our model and not include a site effect.

The model we feel best represents the data is
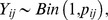
where 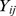 is a binary outcome for *i*th seal at the *j*th time and 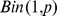 is a binomial distribution with a sample size of 1 and probability parameter *p*,



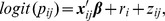
where 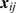 is a vector of explanatory values listed above, 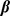 is a parameter vector, *r_i_* is an independent random effect for seal *i* with 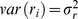, and 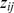 is a temporally auto-correlated random effect for repeated measurements of a seal. The likelihood formed by this model is difficult to maximize, so Wolfinger and O’Connell [31] introduced a pseudo-likelihood approach based on a Taylor series approximation. This method is used in PROC GLIMMIX in SAS and the Splus function glmmPQL (Insightful Corporation, Seattle, WA, USA), which was used by Bengtson et al. [27] for a model similar to ours. Using pseudo-likelihood for our model required inverting an *n_i_*×*n_i_* matrix for each seal, where the number of observations per seal, *n_i_*, was often several thousands. This made the use of commercial software impossible.

We chose an exponential auto-covariance model for repeated measurements,
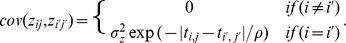
because it can handle missing data and it has an analytical inverse. Here 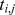 is the time of the *j*th observation for the *i*th seal. We were able to use this autocovariance model, and sparse matrix techniques, to fit models using pseudo-likelihood [28]. We programmed the analysis in the statistical software R [32] and used custom software developed by Ver Hoef et al. [28]. P-values for explanatory variables were based on the Type III hypothesis test as in SAS [33].

We examined p-values for all interaction terms. Following Ver Hoef et al. [34], interactions with *p*>0.15 were removed one at a time, starting with the least significant variable. This step-wise regression continued until the final model structure included all interactions with *p*<0.15. Final statistical significance was based on *p*<0.05. Note that pseudo-likelihood does not use a true likelihood so it was not possible to use AIC. Therefore, we used the traditional approach of stepwise selection of fixed effects based on p-values.

## Results

**Figure 2:**
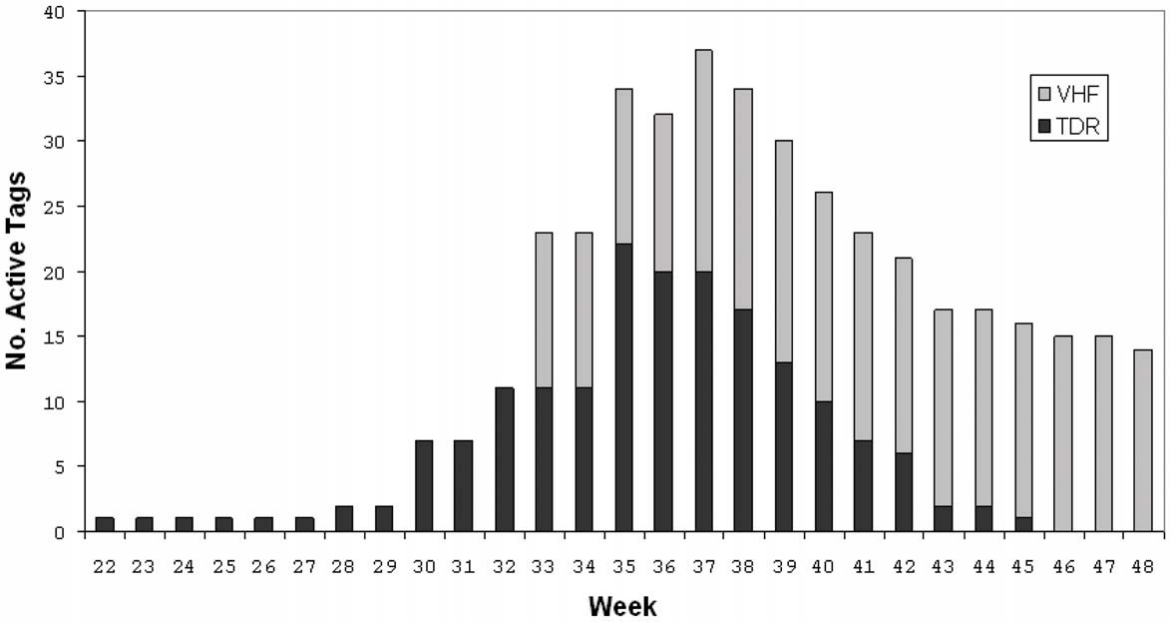
Distribution of recovered (TDR) or deployed (VHF) instruments across weeks of the year (last week of May – last week of November).

The pooled dataset of records from recovered TDRs and VHF deployments spanned three separate years and covered dates from the last week in May through the first week of November (Fig. 2). Of the TDRs deployed on harbor seals within Hood Canal, 25 (12 in 2002 and 13 in 2005) were recovered with data records usable for analysis (Table 1). The distribution of recovered TDRs and deployed VHF tags across age classes (Table 1) was balanced and the number of males and females included in the analysis was identical. The combined TDR and VHF record-set represented 61,430 seal-hours of data.

**Table 1:** Numbers of recovered (TDR) or deployed (VHF) instruments across age and sex classes.

	*Males*			
Year	Adult	Sub-Adult	Yearling	Pup
2002	7	–	–	–
2005	1	3	2	–
2006	1	3	2	2
Total	9	6	4	2
	*Females*			
Year	Adult	Sub-Adult	Yearling	Pup
2002	5			
2005	6	3		
2006	5		3	1
Total	16	3	3	1
TOTAL	25	9	7	3

Six terms were included in the GLMM and we used type III hypothesis testing to determine relative importance within the model. ‘Hour of day’ (*p*<0.001), ‘minutes from high tide’ (*p*<0.001), ‘days from 15 August’ (*p*<0.001) and year (*p*<0.001) were all found to be significant factors (Table 2). Both the sex and age terms were not found to be significant (*p*>0.15) and were, therefore, not included in the final model. The haul-out probability was highest in the ‘minutes from high tide’ categories represented by 33, 65 and 97 minutes after high tide. The coefficients for these categories (1.11524, 1.11693, and 1.11370 on the logit scale) were equivalent and they confirmed previous observations of Hood Canal being a high tide haul-out area. The coefficients for the year term (2002: intercept, 2005: 0.50335 (*p* = 0.095), and 2006: −0.60428 (*p* = 0.034)) suggests the observed year effect was due in large part to a significant increase in haul-out probability in 2005. For further examination of the year effect, values for the other factors were standardized to the adult females, 65 minutes after high tide. We calculated estimates of haul-out probability for each year at 0 and 12 hours on 22 September (Table 3). This analysis indicated a 40–50% increase in haul-out probability in 2005 compared to 2002. In addition to the specific temporal, environmental and physiological covariates, we also investigated interactions between the terms (e.g., hour : minutes from high, days from 15 August : minutes from high). Of the examined interactions, only ‘hour : days from 15 August’ (*p*<0.001) was found to be significant (Table 2).

**Table 2:** Type III hypothesis table for the five terms selected for the final model.

Effect	Num.df	Den.df	F Value	Prob. F
Hour of day	23	61,430	11.81	<0.001
Minutes from high tide	29	61,430	49.87	<0.001
Days from 15 August	1	61,430	306.65	<0.001
Year	2	35	7.84	<0.001
Hour : days from 15 August	23	61,430	13.11	<0.001

‘Num. df’ refers to the number of degrees of freedom and ‘Den. df’ refers to the denominator degrees of freedom.

**Table 3:** Changes in haul-out probability across year (and exposure to killer whale predation) for noon and midnight.

Year	Hour of Day	Haul-out Probability
2002 (Pre-killer whales)	12	0.161
2005 (Post-killer whales)	12	0.240
2006 (2 y w/out killer whales)	12	0.095
2002 (Pre-killer whales)	0	0.344
2005 (Post-killer whales)	0	0.465
2006 (2 y w/out killer whales)	0	0.223

Calculations were standardized on 15 August and 65 minutes after high tide.

**Figure 3:**
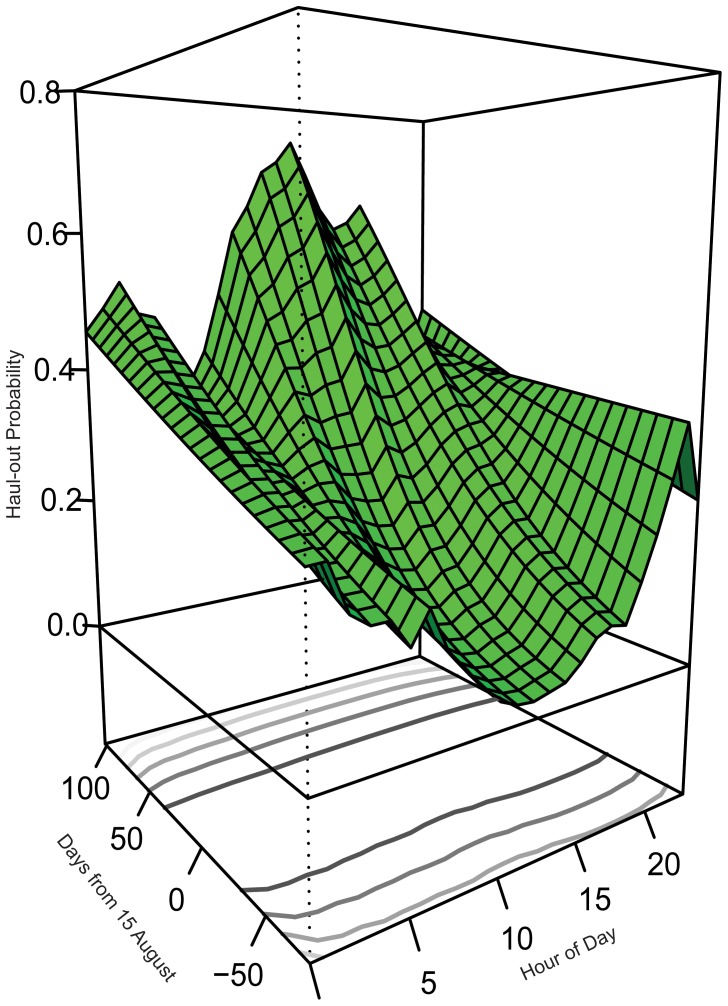
Haul-out probability surface for harbor seals in Hood Canal showing the interaction of ‘days from 15 August’ and ‘hour of day’. Minutes from high was set at +65 and values for sex, age and year were averaged. Contour lines on bottom panel represent a gradient of standard errors for predicted values.

**Figure 4:**
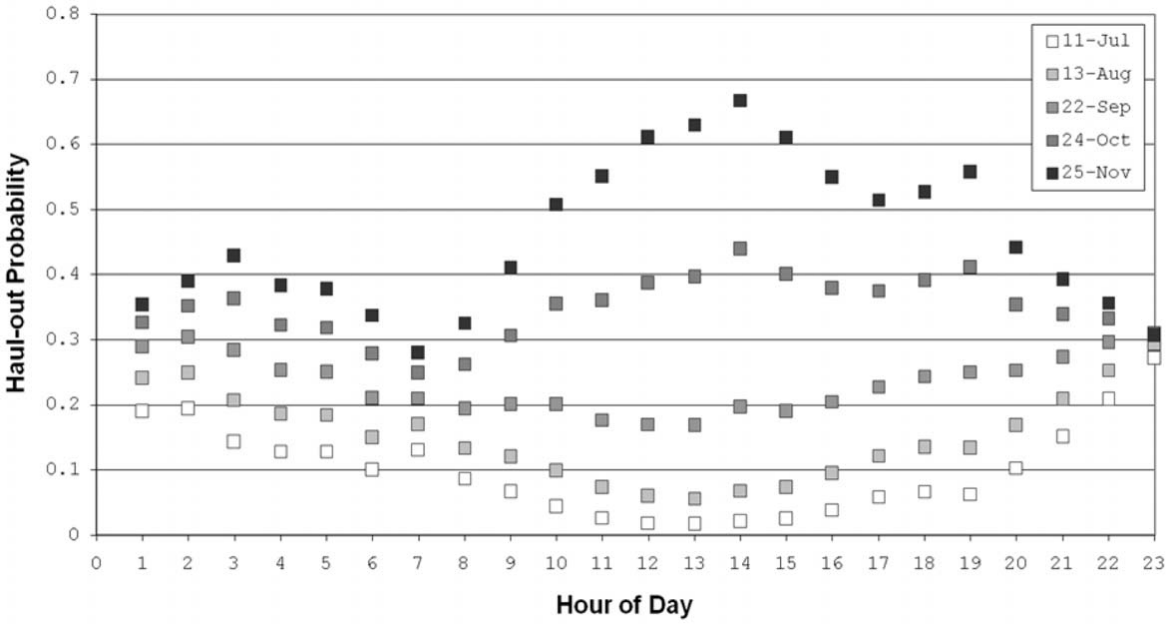
Cross-sectional chart of haul-out probability for five dates from Figure 4 for each hour of the day.

A probability surface (Fig. 3) illustrating the interaction between ‘hour of day’ and ‘days from 15 August’ was created by averaging the effect for year, age and sex while using the middle maximum probability category for ‘minutes from high tide’ (+65 minutes). Predictions from the model (Fig. 4) indicate that at the beginning of the study period (June-August), harbor seals in Hood Canal were more likely to haul out during the night-time hours. As the days progressed into September, there was a linear increase in haul-out probability during mid-day hours. Finally, by late October and November, seals had the highest probability hauling out during the mid-day and afternoon hours.

The estimated autocorrelation was 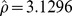. Most of the autocorrelation disappears beyond 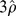 (often called the practical range [35]), which is between 9 and 10 days. Also note that 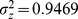 and 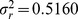, indicating that the extra variation (beyond binomial variance) associated with repeated measurements of a seal is almost twice that of the variation among seals.

## Discussion

The haul-out behavior of harbor seals in Hood Canal differs from that observed at other locations. The association with high tide (as opposed to low tide) was known from previous work, but this study further specifies the relationship and demonstrates the highest haul-out probability occurs during the 1.5 hours after high tide. This effect is likely tied to the nature of the haul-out habitat these seals are using. The tidal sloughs are deep and narrow. Seals cannot access the bank until the water is at or near high tide. These results suggest that once the seals come ashore, they stay for a few hours and leave before the water level drops too low.

Our analysis found no effect of sex or age. This is surprising given our deployments were reasonably balanced across the factors and that our study period encompassed pupping, weaning, breeding and molting. From previous studies of harbor seal behavior our expectation would have been to see an increase in haul-out probability for females in August and September followed by an overall increase in haul-out probability in October and November during the molt. That we did not see an effect of age and sex is likely a combination of low sample size and the higher stringency of a model that appropriately accounts for temporal auto-correlation. We have no corroborating data to provide a biological explanation for no effect of age and sex. With higher sample sizes we would still expect to see differences in haul-out behavior between age and sex classes.

The interaction between ‘day of year’ and ‘hour of day’ is a striking result and suggests the haul-out behavior of harbor seals in Hood Canal is more dynamic than previously thought. Grigg et al. [36] observed seals at Castro Rocks in San Francisco Bay with a more nocturnal haul-out pattern similar to that seen with the Hood Canal dataset during the summer months. Seals at Castro Rocks are exposed to high levels of human activity and Grigg et al. [37] concluded the seals had shifted to a nocturnal haul-out pattern in order to avoid disturbance.

The sensitivity of harbor seals to disturbance has been previously documented [1], [37], and all of the index haul-out sites within Hood Canal experience human disturbance on a regular basis. The haul-out site used in Quilcene Bay is on operational salmon net-pen floats and oyster rafts. These facilities are visited for maintenance or harvest on a regular basis. The Dosewallips haul-out area is contained within the Dosewallips State Park and the tidal sloughs are popular spots for kayakers and canoers to explore. Motorized boats are also in relatively close proximity as they come and go from a nearby marina. The Hamma Hamma site is a working oyster farm. Lastly, the Skokomish site is in close proximity to a kayak rental facility and is a regular spot for tribal and commercial fisheries. The seasonal aspect of human disturbance also correlates well with observed behavior. Recreational activities (e.g., kayaking, pleasure boating) are higher in the summer months with a noticeable drop-off after the Labor Day holiday (first part of September). Most schools return from the summer holiday just before or soon after Labor Day. In response to this reduced activity many businesses reduce hours. The haul-out probability surface shows during late summer, seals are less likely to haul out during the mid-day period. As human activity declines over September, seals are more likely to haul out during the mid-day hours. By October and November, when human presence is reduced, seals are exhibiting a more typical diurnal pattern with the highest probabilities occurring in the mid-day to afternoon hours.

The increase in seals onshore with respect to day of year is not entirely a result of reduced human activity. Seals in Hood Canal have been observed to undergo molt between September and November. Our capture data and deployment lengths for the TDRs (which were adhered to the hair) confirm the timing of molt; the majority of tags fell off between mid-September and mid-October. A few tags detached from the seals in late August and one tag remained on an animal through the first week of November. Given the timing of molt, it is difficult to determine the relative importance of molt versus human disturbance in the observed pattern. It is likely the change in behavior from a predominately nocturnal to diurnal haul-out pattern is related to decreases in human activity, whereas the overall increase in proportion of seals ashore is a response to the energetic demands of molting.

Whereas the haul-out behavior of harbor seals varies within a day and within a year, the behavior is expected to be relatively stable across years. Our results indicated a significant increase in haul-out probability in 2005 compared to 2002 and 2006. While the year effect is potentially confounded by site, such an increase is consistent with the idea that seals exposed to killer whale predation would spend more time ashore to avoid predation. For other harbor seal populations, killer whale predation is likely a regular factor influencing their behavior [24]. Additionally, the normally transient behavior of mammal-eating killer whales precludes them from having a dramatic impact on overall haul-out behavior. The unique situation in Hood Canal was ideal for observing a dramatic shift in behavior. Naive seals were exposed to an unprecedented level of predation over 2.5 years. Consequently, their haul-out behavior underwent a dramatic shift. Our deployments in 2005 began only a few weeks after the whales left Hood Canal. By the time of our 2006 deployments, whales had been absent from Hood Canal for more than a year, and the haul-out probabilities appear to have returned to pre-killer whale levels.

There was significant autocorrelation in these data that ranged up to 10 days. It was important to include autocorrelation in our model because otherwise, statistical inferences, such as the p-values that indicate significance, tend to be too low [34]. This could have caused us to falsely declare an explanatory variable as significant more often than we should.

The extra-binomial variation due to the repeated measures random effect with autocorrelation and the among-seal-variability indicated that it is important to have many seals and a fairly long time series per seal in order to estimate the effects of explanatory variables with much precision. It is especially important to have multiple seals to investigate factors that only change from seal to seal; in particular, sex and age. Note that none of the among-seal factors were significant, whereas all of the within seal factors were highly significant. Much of the time and expense is involved with capturing individual seals; however, future studies would benefit from having more seals when investigating subject-level effects.

Without an appropriate model for haul-out behavior of seals in Hood Canal, it is not surprising that acute population changes due to killer whale predation in 2002 and 2005 were not detected from raw counts [25]. Correcting survey counts within Hood Canal using the GLMM presented here may provide annual population estimates that are more in line with expectations from bio-energetic models. The importance of understanding the haul-out behavior is not limited to evaluating the impact of killer whales. Additional ecological concerns over seal and salmon interactions [25] and low dissolved oxygen events within Hood Canal require adequate monitoring of populations on a localized scale. The model presented here should provide researchers and managers with improved ability to monitor seal population trends and abundance in Hood Canal.

Our results indicated a higher level of plasticity in haul-out behavior for harbor seals than previously described. This contrasts with recent studies showing relatively consistent haul-out patterns across sites and years [17], [18]. This is partly due to the unique opportunity Hood Canal provided. Our deployments spanned a dramatic ecological event that occurred during an extended visit by mammal-eating killer whales, and we were fortunate to recover a large sample of tags that recorded detailed information over a long time period. However, the observations in Hood Canal do provide key insights into harbor seal behavior and have implications for the conservation and management of other populations.
